# Effect of modified waste introduction methods over short-term and long-term use of onsite sanitation systems

**DOI:** 10.1038/s41598-023-35110-x

**Published:** 2023-05-25

**Authors:** L. Astete Vasquez, N. Mladenov

**Affiliations:** 1 Department of Civil, Construction, and Environmental Engineering, San Diego State University, San Diego, USA; 2 Department of Mechanical and Aerospace Engineering, University of California, San Diego, USA

**Keywords:** Civil engineering, Sustainability, Environmental impact

## Abstract

Insufficiently treated wastes contained within onsite sanitation systems (OSS) commonly used by disadvantaged and developing communities contribute to public and environmental health concerns, calling for practical alternative solutions. At the basic level, an improved understanding of the evolution of chemical and physical constituents under different waste introduction methods and for short-term and long-term operation is needed. While receiving non-dilute waste under mixed, unmixed, toilet paper exclusion, and urine diversion (UD) regimes, self-flushing OSS simulated using anaerobic digesters (ADs) were compared during three operational stages: (1) 0–1 month service for unsheltered encampments; (2) 1–3 month disaster relief scenario; and (3) ≥ 3 months representing refugee camps and long-term household use. Although stratification was found to promote suitable conditions for short-term use of self-flushing toilets, mixing increased beneficial biodegradation of organic constituents. Urine-containing ADs demonstrated a shift from sulfide to ammonia odor accompanied by high pH (> 8) after ~ 240 d. *E. coli* reduction following elevated nitrogen and dissolved solids levels pointed to decreased pathogen survival in ADs with urine. The benefits of bacterial disinfection, reduction of sulfurous odors, and heightened organics degradation in mixed, urine-containing ADs suggest this format as more desirable for prolonged use of self-flushing OSS over unmixed or urine-diverting formats.

## Introduction

Despite continued progress toward accomplishing the United Nations Sustainable Development Goal to provide adequate and equitable sanitation for all, 3.6 billion people remained without access to improved sanitation systems as of 2020, almost half of which were left without any form of basic sanitation at all^1^. Socioeconomic constraints, including a lack of government funding and limited local resources, have contributed to the continued use of basic forms of onsite sanitation systems (OSS) such as pit latrines, composting toilets, and aqua privies. These OSS are generally considered more of a form of storage for fecal sludge than a means for treatment, impeding progress toward solutions for their existing shortcomings that link them to public and environmental health concerns^2–4^. Furthermore, OSS deployed for use in disadvantaged circumstances rarely include features desirable to the user, which can hinder their initial acceptance and contribute to operation and maintenance issues that lead to system failure and ultimate abandonment^4–7^. Reducing the number of people without access to safe fecal waste disposal requires efforts to determine practical and holistic solutions for improving existing sanitation systems and devising new approaches to minimize complications associated with their use. To achieve this, developments in OSS should be focused on establishing low-cost^8^, hygienic systems that are user-friendly and sustainable in terms of the system longevity and their potential for water conservation and resource recovery.

Several variations of OSS have been used for the storage and treatment of human fecal waste, each subject to their own set of advantages and disadvantages—the latter of which may be mitigated through practical modifications to system use. Common implementation of the latrine format has continued due to its low construction costs, ease of operation, and accessibility in areas where centralized or decentralized sewage networks are not a viable option^2–5^. While anaerobic processes occurring within latrines provide beneficial stabilization of organic matter^9–11^ and in some cases can contribute to inhibition of pathogenic bacteria^12–15^, contents of traditional latrines are nonhomogeneous, causing internal processes to be performed at uneven rates^11,16^ and resulting in end products of unknown stability and sterility. However, mixing of fecal waste stored in OSS may increase its level of stability, as evidenced by a Jegede et al. study which demonstrated higher treatment efficiency in anaerobic digesters (ADs) systems under mixed conditions^17^—the implications of which are directly translatable to OSS, as demonstrated by a septic tank study by Almomani^18^. Another concern regarding the use of OSS is that nutrient levels from human fecal waste become concentrated over extended periods of use, which pose environmental risks in the occurrence of leaks or contact with groundwater^9,13^. A significant portion of nutrient loads can be sequestered through the application of urine diversion (UD), which is facilitated through fabricated toilet bowls such as EOOS Urine Trap^19^ that separate urine at the user interface. UD has been widely studied for its ability to increase nitrogen recovery and reduce instances of eutrophication^20^, while also reducing energy needs during additional treatment^21^, where necessitated. Other issues associated with the use of pit latrines are their potential to leach contaminants into groundwater^3^ and tendency to cause insect nuisances, which may both be mitigated by implementing water-tight, container-based OSS^2,22^.

This study assessed the evolution of wastewater composition in unsewered, self-flushing, container-based toilet systems, simulated by bench-scale anaerobic digesters (ADs) that incorporated a plunging mechanism to promote circulation of digested contents for ‘flushing.’ Despite the worldwide use of waterborne onsite sanitation systems and extensive studies on systems such as septic tanks, pour-flush latrines and aqua privies, there are still gaps in understanding of fundamental processes occurring in OSS under scenarios of mixing, stratification, and different sanitation practices, especially in response to non-dilute waste introduction. The results of this study were expected to discern whether mixing of OSS contents provides advantages for treatment of fecal waste over traditionally static latrines, and whether other sustainable or culturally normative sanitation practices, such as urine diversion or separate discarding of toilet paper, significantly impact chemical and physical characteristics of the effluent. The information gained from this study should facilitate further development of improved OSS and could inform decisions surrounding the application of similar modifications in future systems.

## Materials and methods

### Waste products

Dog feces were introduced to the ADs to simulate the introduction of non-dilute human excreta to OSS. The use of dog feces for simulating human fecal waste in this study was expected to have significant advantages over previous AD and sanitation systems studies, which have used cow, swine, and chicken manure, or laboratory-formulated simulant wastewater^5,15,17^. In addition to the benefits of using real waste to achieve realistic fecal composition and consistency, a study by Coelho et al. concluded that the canine fecal microbiome more closely resembles that of humans^23^ in contrast to that of other mammals, implying that microbial organisms introduced to the ADs could resemble those encountered in OSS used by humans. The dogs’ dry food diet was supplemented with a stew containing grains, vegetables, and meat, along with periodic feeding of raw vegetables. This omnivorous diet reflects components of the human diet in ways that cannot be met by excreta from farm animals, which typically consists of grains and grasses. Samples of fresh canine feces were collected and stored in air-tight containers at 4 °C and used within one week following collection. Plant debris and soil grit were removed as feasible prior to introduction to the ADs, and feces were divided into 4–6 small portions during introduction.

Other waste products used for this study include formulated synthetic urine and commercial-grade toilet paper. The use of simulant urine reduced obstacles with collection and offered a stable product for replicable results^24^. Synthetic urine was prepared in accordance with methods defined by Pronk et al. which includes urea (16.2 gL^−1^), NaCl (6.2 gL^−1^), KCl (4.7 gL^−1^), NaH_2_PO_4_ (3.9 gL^−1^), Na_2_SO_4_ (2.8 gL^−1^), and NH_4_Cl (1.8 gL^−1^) dissolved in demineralized water, adjusted to pH = 6 using HCl and NaOH^25^. This mixture was stored at 4 °C for up to one month. Toilet paper was retrieved from a campus bathroom, provided by Kimberly-Clark Corporation.

### Experimental setup

Four anaerobic digesters (ADs) were modeled after the Gendarme Sanitation System (Supplementary Fig. S1), which is a water-filled, container-based, self-flushing OSS used in southern Africa that employs anaerobic digestion of concentrated blackwater. The ADs were constructed from 2 L HDPE jars, tubing for effluent (supernatant), and fabricated plunging mechanisms, where applicable (Supplementary Figs. S2 and S3). The ADs were fed, operated, monitored, and sampled for 725 d, as described below.

The ADs were initially filled with tap water before 15 mL of feces macerated in a low volume of tap water were introduced as a starter feed. After one week, 4 g of feces, 30 mL of synthetic urine, and 0.1 g of commercial grade toilet paper were added twice weekly in accordance with waste introduction schemes shown in Table 1. Waste volumes introduced to the ADs were based on the fecal quantities produced per capita per day as detailed by WHO^26^, average urine production from Rose et al.^27^, and average toilet paper usage estimated by Toilet Paper History^28^, all of which were proportionally scaled to the size of the bench-scale ADs in comparison to the 250 gal drums typically utilized for a family of 4–6 users (for waste quantity calculations, refer to Supplementary Information Sect. 1.2). Following each waste introduction, plunging mechanisms for mixed digesters were pumped 40–60 times to promote homogenization of the water column at the rate expected from regular daily use of OSS.

**Table 1:** Feeding and operational schemes for ADs.

	Mixing	Feces	Paper	Urine
Mixed (MX)	**•**	**•**	**•**	**•**
Unmixed (ST)		**•**	**•**	**•**
Urine diversion (UD)	**•**	**•**	**•**	
No toilet paper (NO TP)	**•**	**•**		**•**

### Water quality analyses

Electrical conductivity (EC) and pH were measured within the AD supernatant layer (1.5–5 cm depth) every other week using a Fisherbrand Accumet AP85 portable waterproof meter. Samples of supernatant were collected monthly for analysis of solids, chemical oxygen demand (COD), nutrients, and dissolved organic carbon. Total suspended and dissolved solids (TSS, TDS) were separated using 1.2 µm glass filters prior to drying and combusting in accordance with APHA^29^ gravimetric methods, and a portion of filtrate was used for nutrient analysis. HACH Test n’ Tube reagent vials and a DR3900 spectrophotometer were used to quantify COD, total ammoniacal nitrogen (TAN), nitrite-N (NO_2_^−^), nitrate-N (NO_3_^−^), and total phosphorus (P_tot_). Samples were filtered through 0.7 µm syringe filters prior to dissolved organic carbon measurements using a Shimadzu TOC-L Total Organic Carbon and Total Nitrogen Analyzer, which employs a high temperature combustion method. In addition to supernatant, settled sludge was collected periodically from the bottom layer of ST for the enumeration of *Escherichia coli* (*E. coli*), total coliforms and total heterotrophs, which were performed using IDEXX Colilert-18, Colisure, and HPC for Quanti-Tray methods. Samples were diluted as necessitated by analysis detection limits using deionized water. Dissolved oxygen was measured at ~ 5 cm depth using a YSI ProODO optical DO meter.

### Aerobic biodegradability test

A biodegradability test was performed twice in accordance with methods detailed in Bakare et al.^11^, which quantifies the amount of biologically-oxidizable organic matter in fecal sludge samples and points to the degree of stabilization achieved within the digester. 50 g of mixed digester contents (and 50 g each of supernatant and sludge from ST) suspended in 1 L of tap water were added to 2 L volumetric flasks, and COD was recorded for the mixture before and after 5 d of aeration. COD lost during aeration is considered the biodegradable portion of the COD content, expressed as g biodegradable COD/g COD (biodegradability)^11^.

### Comparison of introduction methods and operational stages

Biochemical changes were characterized under four scenarios of non-dilute fecal waste introduction: (1) mixing (MX) after waste introduction using a plunging mechanism to increase microbial contact to available substrate within ADs^17,18,30^, (2) unmixed OSS operation (static = ST) with stratification occurring between the sludge layer and liquid supernatant, as is common in septic tank and aqua privy systems, (3) mixing with exclusion of toilet paper (NO TP), which may lower the C:N ratio, affecting bacterial activity and ammonia inhibition^31,32^, and (4) mixing with urine diversion (UD), which may affect pH and reduce ammonia inhibition in ADs^5^. The Data Analysis tool in Microsoft Excel for Mac version 16.63 was used to determine whether different introduction methods (all, mixed, urine-containing, and individually compared) demonstrated statistically significant similarities. Statistical analyses included analysis of variance two-way (ANOVA, Supplementary Table S1) at a confidence interval of 95% (*p* ≥ 0.05) for concentration values, and Spearman’s correlations (Supplementary Table S2) to compare concentration trends, over the 725 d study period.

Provision of adequate sanitation services is an issue of primary concern in scenarios regarding unhoused citizens, disaster relief efforts, refugee camps, and households in rapidly developing, low-income communities—each of which may necessitate use of OSS over differing lengths of time. Evaluating the quality of AD contents during defined periods of use allows for the interpretation of best waste introduction practices for a variety of real-world applications. As such, changes to wastewater were also evaluated in response to three stages of operation: Stage 1 (0–1 month) simulates short-term applications such as mobile trailer units for unhoused community members that are emptied regularly; Stage 2 (1–3 months) represents temporary disaster relief scenarios; Stage 3 (≥ 3 months) simulates extended durations of use, including refugee camps and onsite sanitation technologies for single family dwellings.

## Results and discussion

### Physical and performance characteristics

Physical characteristics of AD digestate and performance impedances experienced over the 725 d study can potentially imply increased maintenance needs and consequences upon OSS longevity during full-scale use. Without the introduction of liquid contents, UD required periodic refilling with tap water (~ 3% total volume each month) to maintain the desired surface level for sustainable operation, which could pose limitations in water-scarce regions. The plunging mechanism of UD frequently became clogged, demonstrating an apparent deficiency in digestion of solids within the tank, which could contribute to failure of self-flushing mechanisms. NO TP experienced the least operational issues, and effortless use of the agitator pump occurred without clogging. Contents of ST became stratified, resulting in a thick sludge bottom layer and a clear supernatant that became slightly viscous and rust-colored over time (Supplementary Fig. S4). The separation of layers in ST could remove the risk for clogging self-flushing mechanisms and could be easily achieved through implementation of a baffled chamber that separates the plunging mechanism from a settling chamber in instances where separate discarding of toilet paper is less practicable.

Adoption of OSS and sufficient user satisfaction may be impacted by visual and olfactory changes to the ADs that were observed during this study. Approximately 240 d into operation, a noticeable shift from sulfurous to ammonia odor occurred in ADs receiving urine. The ammonia odor, which is reminiscent of cleaning solution odors, may be more desirable for users of OSS. By contrast, UD maintained a foul sulfurous and earthy odor throughout the experiment, which was exacerbated by effervescence that released unpalatable gases to ambient air. UD also experienced frequent fungal growth at its air-exposed surface (Supplementary Fig. S5), presumably due to reduced levels of ammonia within this AD. A similar fungal growth response was shown by Ellouze et al., where performance of white rot fungus in an AD used to treat landfill leachate was unimpeded up to 2 gL^−1^ NH_3_-N but was inhibited at concentrations above 5 gL^−1^ NH_3_-N^33^. Based on this evidence, UD is less desirable for long-term use unless intentional modifications to pH would lend equal results concerning odor and fungal growth.

### Chemical and bacterial characteristics

Chemical and bacterial characteristics of the AD supernatant were heavily influenced by the exclusion of urine, with UD demonstrating lower pH, EC, nutrients, COD and dissolved organic carbon, while also exhibiting higher concentrations of *E. coli*. Similarities between MX and NO TP implied insignificant differences caused by the absence of toilet paper. Mixing of ADs resulted in higher pH and *E.coli* than ST, and lower nutrients, COD and dissolved organic carbon, indicating that organics were more efficiently degraded when mixed, and implying reduced needs for further biological treatment of OSS contents. Due to the similarity between all ADs during short-term (≤ 3 mo.) operation, ADs without mixing are recommended for their simplicity to implement, in addition to their ability to minimize risks for exposure to pathogens and reduce effluent pH. Qualities of mixed AD supernatant including lower COD, dissolved organic carbon, and nutrients that developed after 6 months demonstrated the benefits of mixing over unmixed OSS for extended periods of usage, particularly in instances where UD is less practicable.

**Figure 1:**
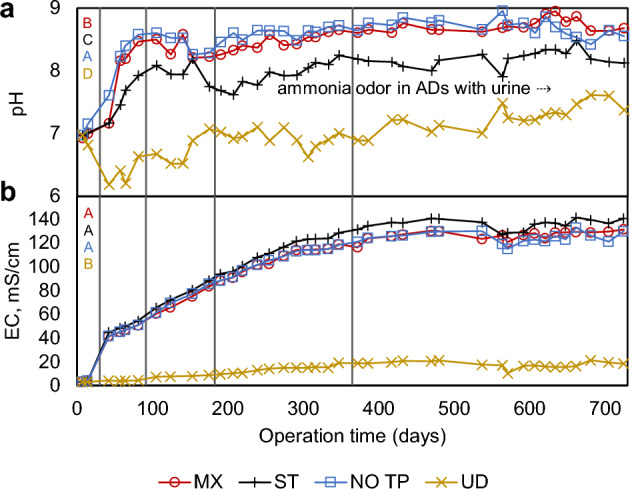
Chemical properties of AD supernatant in response to non-dilute waste introduction. (**a**) pH and (**b**) electrical conductivity (EC) measured over 725 d of operation. Plots are divided at days 30, 91, 183 and 365 to mark stages of operation. Letters at top left denote ANOVA pairing (*p* ≥ 0.05) over 725 d. Text in A represents a shift from sulfur to ammonia odor in urine-containing ADs at ~ 240 d.

The ADs were maintained at ambient temperatures ranging from 21 to 24 °C, and the maximum dissolved oxygen concentration was 0.13 mgL^−1^. As shown in Fig. 1A, pH changed dramatically during Stages 1–2 of operation for all ADs and demonstrated generally stable values after 183 d in the range of ~ 7–9 (MX = 8.6 ± 0.17, ST = 8.1 ± 0.20, NO TP = 8.7 ± 0.12, UD = 7.1 ± 0.25 ≥ 183 d), the differences of which can be attributed to several factors. For instance, ST exhibited lower pH than other urine-containing ADs that were mixed, suggesting that organic decomposition was inhibited during digestion under unmixed conditions. Mixing has shown to increase microbial access to organic substrates^18,34^, leading to their solubilization, and resulting in products such as ammonia^35,36^, carbonate, and bicarbonate^37^ which contribute to elevated pH levels^38^ that have been associated with a higher degree of disintegration in fecal waste^39^. While ADs ideally operate at pH values of 6.8–7.4^40^, pH > 8 has been observed in the treatment of wastes high in proteins^35^, and has also been associated with wastes containing urea^21,41^, which was supported by high correlations between pH in ADs with urine (ρ = 0.784–0.923). While pH in UD, which remained circumneutral for much of the study period, eventually reached values within the optimal range for operation of ADs, its slow progression may impact the process of methanogenesis^35,39,40^, which limits the accumulation of acids responsible for most AD failures^41^. This suggests that the inclusion of urine may facilitate AD processes during early stages of OSS operation for systems planned for use ≥ 6 months.

Gradual increases in EC (Fig. 1B) were observed through the first 12 months of operation, after which point values plateaued (MX = 126.6 ± 3.7, ST = 135.8 ± 4.2, NO TP = 124.7 ± 4.4, UD = 17.8 ± 2.6 mS/cm ≥ 365 d), potentially due to supersaturation of the soluble anions and cations in the AD supernatant^42^. EC was distinguishably lower in UD due to the exclusion of ionic compounds associated with urine^43^. ADs containing urine maintained similar EC concentrations (*p* = 0.31) and demonstrated very highly correlated trends (ρ = 0.910–0.997), suggesting that mixing and toilet paper exclusion had no significant impact on their accumulation of salts and inorganic compounds^21,27^.

**Figure 2:**
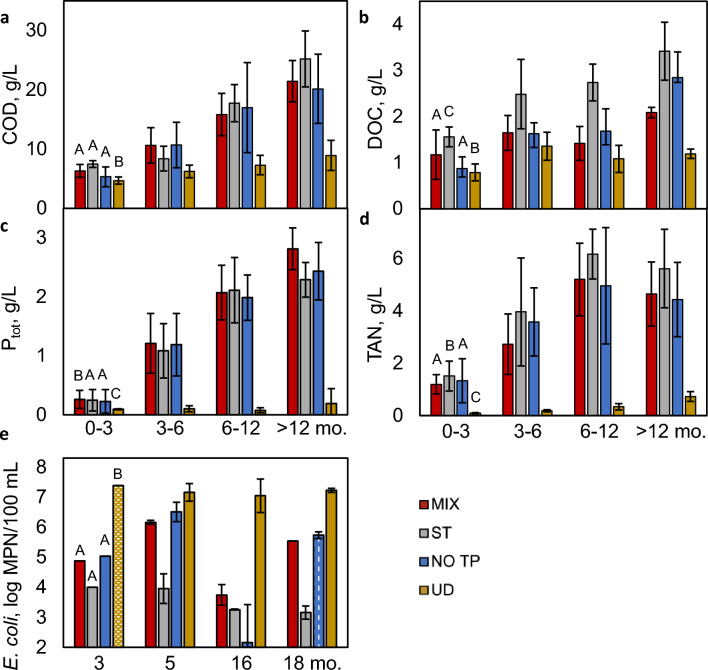
Composition of AD supernatant in response to non-dilute waste introduction. Average measured values (± standard deviation, error bars) for (**a**) chemical oxygen demand (COD), (**b**) dissolved organic carbon (DOC), (**c**) total dissolved phosphorus (P_tot_), and (**d**) total ammoniacal nitrogen (TAN = NH_3_ + NH_4_^+^) over 681 d of operation, divided into operational stages. Letters above first column cluster denote ANOVA pairing (*p* ≥ 0.05) for data obtained over 681 d. (**e**) Log_10_ concentrations of *E. coli* in digester supernatant on days 98 (3 mo.), 164 (5 mo.), 482 (16 mo.), and 551 (18 mo.). Pattern fill for *E. coli* concentrations in ST during month 3 (horizontal) represents a value that was below the measuring range (< 1 MPN/100 mL at 10^−3^ dilution), and NO TP during month 18 (vertical) was over the measuring range (> 2,419.6 MPN/100 mL at dilution of 10^−2^).

Dissolved organic carbon and COD both increased over time and at different rates between all ADs during Stages 1 and 2, as shown in Fig. 2A,B. As urine contributes 10–27% of COD introduced to wastewater^27,43^, low COD concentrations observed in UD (8.9 ± 2.5 gL^−1^ ≥ 365 d) were expected, along with similarities (*p* = 0.06) between ADs with urine. COD is also influenced by insoluble toilet paper cellulose^44,45^, which may be responsible for the slightly lower concentrations observed in NO TP, which were 97.3 ± 0.3% concentrations found in MX (MX = 21.4 ± 3.5 gL^−1^, NO TP = 21.2 ± 5.8 gL^−1^ ≥ 365 d). COD was eventually the highest in ST (25.2 ± 4.7 gL^−1^ ≥ 365 d), which may further indicate lower degradation of organics in OSS without mixing^18,46^. Dissolved organic carbon concentrations (MX = 2.1 ± 0.1, ST = 3.4 ± 0.6, NO TP = 2.8 ± 0.6, UD = 1.2 ± 0.1 gL^−1^ ≥ 365 d) were eventually highest in ST despite similarities between ADs containing urine (*p* = 0.28 for MX and NO TP) and were lowest in UD, demonstrating a stark contrast between the degradation of organic materials occurring under unmixed conditions (low) and in the absence of urine (high)^46^.

Nutrients (P_tot_ and TAN, Fig. 2C,D) increased in all ADs in response to the continued introduction of waste and accumulated at a higher rate during Stages 1–2 than Stage 3. Nutrient concentrations were highly correlated in ADs with urine (P_tot_ ρ = 0.939–0.957, TAN ρ = 0.838–0.992), and averaged ≥ 88.5% higher concentrations than in UD, resulting from the addition of urine which contains the largest fraction of nitrogen and phosphorous associated with fecal waste^27,47^. MX (P_tot_ = 2.8 ± 0.2 gL^−1^ ≥ 365 d) contained higher concentrations of P_tot_ than ST and NO TP (*p* = 0.19, 2.3 ± 0.3 and 2.4 ± 0.5 gL^−1^ ≥ 365 d, respectively), suggesting that the absence of toilet paper in the supernatant could potentially reduce phosphorous in the effluent, which can be achieved either through settling or exclusion. Once the elevated pH levels in ADs with urine reached stable levels, TAN concentrations fluctuated (~ 255 d), and dramatic increases in TAN discontinued (MX = 4.6 ± 1.2, ST = 5.6 ± 1.5, NO TP = 4.4 ± 1.4, UD = 0.7 ± 0.2 gL^−1^ ≥ 365 d). This fluctuation can be attributed to pH-dependent NH_3_/NH_4_^+^ equilibrium (Supplementary Eqn. S1)^48^, which causes conversion of NH_4_^+^ to NH_3_ at higher pH (Supplementary Fig. S6) and can result in losses due to volatilization of NH_3_^49^, explaining the development of ammonia odor observed around that same time. TAN was highest in ST, which exhibited a lower pH than the other urine-containing ADs (*p* = 0.65 for MX and NO TP), further supporting the explanation of pH-driven volatilization and NH_3_ loss in other urine-containing ADs. Concentrations of NO_3_^−^ and NO_2_^−^, which ranged from 1.6 to 34.3 mgL^−1^ and 0.11–36 mgL^−1^ respectively, remained low in all ADs under anaerobic conditions. Overall, these results suggest that mixing in the ADs may increase beneficial oxidation of organic materials and slightly reduce TAN concentrations where urine diversion is not applied.

*Escherichia coli* concentrations demonstrated discernable differences between ADs under different conditions, with average values of MX = 5.1 ± 0.9, ST = 3.5 ± 0.3, NO TP = 4.9 ± 1.6, and UD = 6.6 ± 1.4 (log MPN/100 mL) over 551 d. The highest *E. coli* concentrations were observed in UD (Fig. 2E), suggesting reduced viability of indicator bacteria in ADs with urine, possibly due to elevated pH levels, EC, or ammonia concentrations that are less suitable for their survival^14,15,50^. These results point to the potential for beneficial disinfection of *E. coli* within anaerobic OSS containing urine (*p* = 0.28, ρ = 0.998 for MX and NO TP), despite their undesirable accumulation of nutrients. While bacterial concentrations appeared lower under unmixed conditions, concentrations of *E. coli* in the sludge layer of ST, which was sampled at a lower frequency to avoid disturbing its unmixed conditions, were ~ 4-log and 2-log greater than the supernatant on days 164 and 551, respectively, and were higher than the bacterial concentrations in all other ADs on those days. This suggests that bacteria in ST were concentrated within the layer of settled solids and were potentially experiencing lower degradation in the absence of mixing. Due to the high dilutions required to measure bacterial concentrations in the high strength AD supernatant, results for *E. coli*, total coliforms, and total heterotrophs exhibited variability of up to 3.4-log order of magnitude for an individual sample (Supplementary Figs. S7–S9), presenting obstacles for enumeration. To overcome this error while still enabling the illustration of changes to *E. coli* over time, Fig. 2E includes results below or above the measuring range. Concentrations of total coliforms and *E. coli* were approximately equal (total coliforms = 1.1 ± 0.2 times the *E. coli* concentration on average, Supplementary Fig. S7), affirming that *E. coli* were dominant in the ADs. Concentrations of heterotrophic bacteria, ranging from 6-log MPN/100 mL to 10-log MPN/100 mL in all ADs, varied little throughout the study, indicating that conditions within the ADs were not limiting to the survivability of microorganisms that perform breakdown of organics^51^. These results demonstrated that the inclusion of urine in OSS reduces risks for exposure or release of pathogens, both of which are further reduced under static conditions.

### Solids and biodegradability

Solids accumulated in OSS are a result of the waste products introduced over time, and can potentially affect their performance and longevity (clogging, filling), cause detrimental environmental impacts upon release of putrescible organics, and dictate the energy needs and types of treatment used for further polishing to meet environmental targets. AD processes occurring within OSS stabilize organic portions of the waste, producing readily reusable substances for nutrient recovery or direct agricultural application^5,6,9,11^. Some solids discharged from OSS can also provide advantageous barriers to impede the transport of pathogenic organisms^27^. TDS accumulated in ADs receiving urine, and mixing increased TSS within the supernatant while also positively influencing biodegradation of AD contents. The exclusion of toilet paper slightly reduced total solids. These results suggest that different practices (mixing, urine diversion, toilet paper exclusion) could be employed depending on the intended application or length of use.

**Figure 3:**
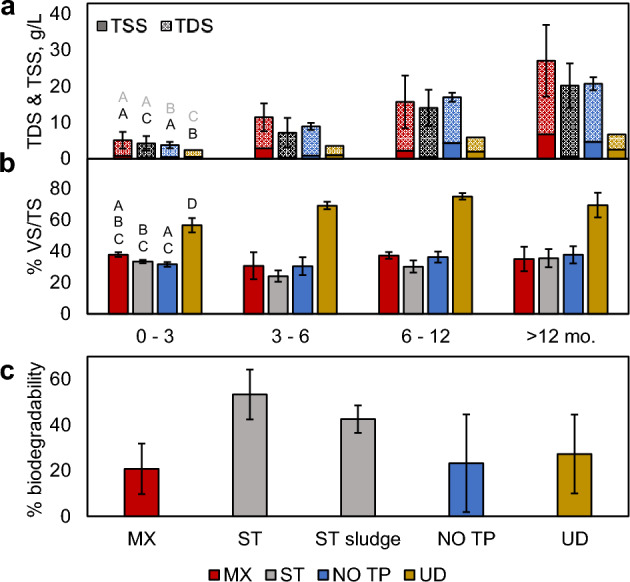
Solids concentrations and aerobic biodegradability for AD supernatant (and sludge, where denoted) in response to non-dilute waste introduction. Average measured values (± standard deviation for total solids = TDS + TSS, error bars) for (**a**) total dissolved solids (TDS, pattern fill) and total suspended solids (TSS, solid fill), and (**b**) fraction of volatile to total solids (%VS/TS). Letters above first column cluster denote ANOVA pairing (*p* ≥ 0.05, gray for TDS and black for TSS in 3b) for data obtained over 681 d. (**c**) % biodegradability (n = 2) in AD digestates on days 706–720.

Total solids were similar between all ADs during Stages 1 and 2, and increased in response to repeat introduction of waste products over 681 d (Fig. 3A). TDS (MX = 20.4 ± 1.2, ST = 19.6 ± 2.3, NO TP = 16.1 ± 1.6, UD = 4.2 ± 0.7 gL^−1^ ≥ 365 d), like EC, remained lower in UD than urine-containing ADs due to the exclusion of salts from urine^21,27,39^, and were most similar between MX and ST (*p* = 0.25, ρ = 0.907–0.966). TSS (MX = 6.7 ± 4.5, ST = 0.6 ± 0.2, NO TP = 4.7 ± 2.1, UD = 2.5 ± 2.2 gL^−1^ ≥ 365 d) was lowest in ST due to stratification of the AD contents, which could be beneficial for periods up to 6 months where degradation is not as important for system longevity, and were most similar between MX and NO TP (*p* = 0.25) due to mixing. The organic portion of solids within the ADs, represented as %VS/TS (Fig. 3B), were relatively unchanged throughout each stage of the experiment due to the continued introduction of volatile fecal solids, with average values of MX = 35.2 ± 6.5%, ST = 32.1 ± 6.2%, NO TP = 35.4 ± 5.4%, and UD = 69.2 ± 7.6% over 681 d, and similarity observed between urine-containing ADs (*p* = 0.06). While urine contributes a significant portion of the VS found in excreta^5,27^, its exclusion and correspondingly reduced TDS led to remarkably higher %VS/TS in UD, with results for VS and TS respectively ranging from 39 to 112% and 24–60% of the average values observed in urine-containing ADs.

ADs that employed mixing had relatively low biodegradability, as reflected by the results of aerobic biodegradability tests conducted on days 706–720 (Fig. 3C). Average biodegradability was highest in ST supernatant and sludge (53.4% and 42.6%, respectively), implying that its contents had undergone the least degree of stabilization prior to sampling^11^. The lowest biodegradability observed in MX and NO TP, at 20.8% and 23.3%, indicated that mixed conditions promoted the breakdown of organic constituents and left behind more recalcitrant organic matter. Biodegradability was also relatively low in UD (27.3%), suggesting that despite having a high organic fraction, the organic matter in UD was similarly susceptible to biodegradation under mixed conditions. While stratification in ST is expected to minimize functional issues caused by suspended solids, it may experience more rapid filling rates as the settled solids resist biological breakdown^18,34,52^. This evidence suggests that mixing of OSS contributes to greater stabilization of organic OSS contents and is therefore preferential for applications planned for ≥ 6 months of use, where it can potentially reduce filling rates and diminish needs for secondary biological treatment to prevent adverse environmental impacts.

### Simulation and replicability

Results from this study were subject to limitations regarding the intermittency of feeding and mixing, detection ranges for employed analysis methods, and reactor setup. While the feeding and mixing rate of 2x/wk is likely to have allowed short periods of stagnation that may not occur during daily use of facilities, noticeably different characteristics between AD contents were observed throughout the duration of the study, suggesting that the intermittency had very low, if any, effect upon the results. Although the highly concentrated AD constituents required large dilutions to obtain results for COD, dissolved organic carbon, nutrients, and bacterial enumeration, accuracy of results was ensured by using multiple dilutions and replicate samples for analysis. In the case of bacterial enumeration, the margin of error was quite high. We hypothesize that even with frequent mixing that three of the reactors received, spatially heterogeneous flocs of organic matter and microbial communities may have formed. Lastly, the configuration of the studied ADs offered easy access to alleviate clogging of internal components for continuation of the experiment, which would be impractical during real use of underground tanks and result in cessation of mixing if large or insoluble materials became embedded in mesh screening.

Despite limitations, similarities were found between the chemical characteristics of ADs used in this study and previous works on pit latrine sludge and anaerobic digestion of various fecal sludges. For example, the pH, COD, and nutrient concentrations of ADs from our study fell within the range of partially-degraded pit latrine sludge observed in the study by Zuma et al.^16^ The pH in our ADs containing urine were also comparable to a study by Colón et al.^5^ on unmixed ADs under continuous introduction of non-dilute simulant excreta, which reached a pH of 8.2, but only in response to elevated ammonia concentrations in the feed solution. COD in all ADs were similar to levels observed in pit latrine sludge following anaerobic digestion by Changara et al.^13^, which also demonstrated similar pH to UD from our study. Lastly, %VS/TS and %biodegradability from our ADs fell within the range of partially-degraded pit latrine sludge detailed by Bakare et al.^11^. These findings both support the validity of results from this simulation and promote future use of the chosen methods for replicable execution of OSS studies.

### Conclusions and recommendations

We assessed chemical and physical changes to non-dilute fecal waste stored within simulated water-filled, container-based, self-flushing OSS over 725 d, which varied in response to different waste introduction methods, impacting their performance and potentially impeding their user adoptability. The separation of sludge and supernatant observed in ST may reduce functional issues caused by bulky organic materials and minimize risks for pathogen exposure, making it more feasible for short-term use (≤ 3 months). However, changes to the supernatant in mixed ADs implied advantageous stabilization of organic matter during long-term periods of waste introduction (≥ 6 months), suggesting that OSS fill rates and environmental impacts caused by high COD and nutrient concentrations may be reduced by mixing OSS contents. While desirable quality of effluent from UD demonstrated multiple benefits for sustainable OSS performance, issues with clogging, odors, and increased *E. coli* survival observed after 3 months made it less user-friendly than urine-containing ADs unless modifications to pH are employed to reduce odors and pathogens. Considering all evidence from this study, it is recommended to employ mixing with traditional waste introduction methods for best results when use of OSS is planned for 6 months or longer.

The diversity of microbial consortia may vary between the waste introduction schemes explored in this study, which has implications for both the internal treatment mechanisms of ADs and their ability to produce biogas for sustainable energy recovery. Data on the biogas potential of standalone OSS is limited, particularly under conditions of mixing, no mixing, and urine diversion. It is therefore recommended for future studies to explore the capability of similar OSS to produce sufficient biogas under various waste introduction and operating conditions, in addition to reporting on distinctions between their microbial abundance. Additionally, the potential degradation of excreted pharmaceuticals or other contaminants of emerging concern under differing non-dilute waste introduction methods (specifically mixed versus unmixed) will contribute to improvements of future and existing OSS for beneficial outcomes regarding public and environmental health.

## Supplementary Information

Supplementary Information.

## Data Availability

Data generated by the current study will be made available upon reasonable request to the corresponding author.
