# Supplementary material for: Effect of modified waste introduction methods over short-term and long-term use of onsite sanitation systems
Source: Sci Rep. 2023 May 25;13:8506. doi: 10.1038/s41598-023-35110-x (PMC10212933; doi:10.1038/s41598-023-35110-x)
Supplement: Supplementary file 1 — Supplementary Information. [file 41598_2023_35110_MOESM1_ESM.pdf]

Supplementary Information

Effect of modified waste introduction methods over short-term and long-term use of onsite sanitation systems

Astete Vasquez, L.[1,2*], Mladenov, N.[1]

[1] Department of Civil, Construction, and Environmental Engineering, San Diego State
[2] University; Department of Mechanical and Aerospace Engineering, University of California, San Diego;
[*] Corresponding author: Tel +1 (602) 910-9960; Email address: Lilith.Astete@gmail.com

Supplemental Text

1. Experimental setup
2. Statistical analysis
3. Characteristics of anaerobic digestate
4. Bacterial characteristics of anaerobic digestate

1. Experimental setup

1.1. Construction of bench-scale reactors

The design of reactors used in this study was inspired by Gendarme Sanitation Solutions (Fig. S1), an onsite sanitation system (OSS) implemented in the southern region of Africa, which includes two identically-constructed agitators for 1) flushing immediately after direct use, and 2) intermittently from the outside for additional mixing. The agitators included in the bench-scale reactors simulated the external agitator pump, which draws mixed contents from the bottom of the tank and creates a showering effect over the surface to wet floating particulates and promote homogeneity of the storage vessel contents. The vessel contains water, and receives cow manure as seed for startup of anaerobic digestion (AD) processes.

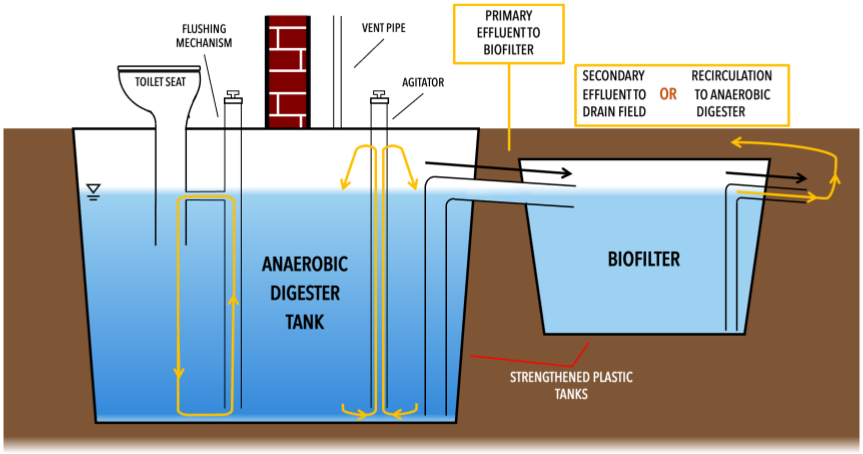

Figure S1. Conceptual overview of Gendarme Sanitation Solutions waterless flushing toilet and anaerobic digester treatment system, including primary anaerobic digester tank with flushing mechanism and agitator pump, and effluent to secondary biofilter.

Bench-scale ADs (Fig. S2) were constructed as described in the Materials and Methods section, using 2 L HDPE wide-mouth packer jars from Fisher Scientific. Their surfaces were wrapped with opaque tape to prevent exposure to UV light during disinfection of the biosafety cabinet used as housing for the experiment (Fig. S3A). The foam lining was removed from the lids during construction, and agitator pumps were modified from children's water shooter toys with t-connectors for tubing as handles. Modifications included a 3 cm notch cut into the bottom exterior to allow space for fluid to enter the column, and holes drilled into the top for a showering effect, illustrated in Figure S3B. Fiberglass window screen material was folded and attached using zip ties to the bottom of the pumps to filter large materials from the column and prevent clogging of the shower holes. The waste introduction methods are summarized in Figure S3C.

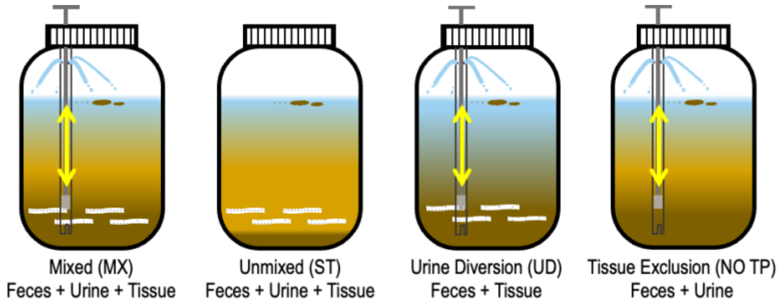

Figure S2. Overview of bench-scale ADs simulating use of OSS and respective waste introduction regimes.

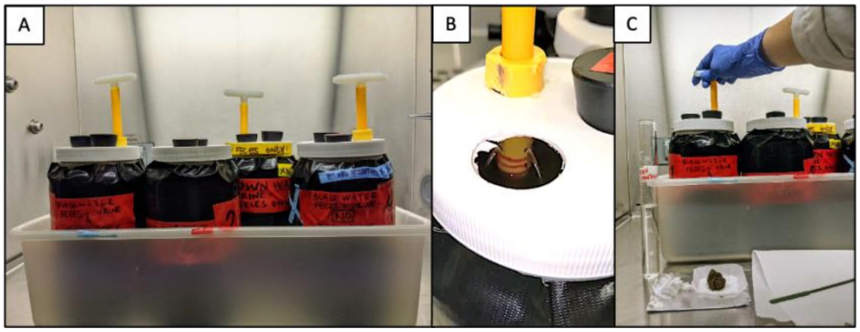

Figure S3. A) Bench-scale digesters with constructed plunging mechanisms, B) view of showering effect from agitator pumps while mixing is performed, C) summary of waste introduction including feces, tissue, synthetic urine, and mixing.

1.2. Waste introduction calculations

The average production of feces and urine varies throughout literature, and is influenced by diet and water consumption[26]. For this study, we assumed 190 g feces/cap·d[25] and 1,420 mL urine/cap·d[26]. Waste introduction quantities to simulate regular use of OSS at the bench-scale were calculated for 4 people (to account for smaller volumes generated by children) and a usage rate of 5x/wk. Toilet paper quantities were calculated assuming the use of 100 rolls toilet paper/cap·yr at 200 g/roll toilet paper[27] (excludes cardboard tube) for 5 people (assuming toilet paper use is not variable across age groups). Quantities were scaled at a rate of 1:473 (2 L digester:250 gal drum), and used a feed rate of 2x/wk.

2. Statistical analysis

2.1. Two-way ANOVA

Two-way analysis of variance was performed using the raw data obtained from analyses used to characterize changes to the AD supernatant from this study. This enabled us to assess significant differences ($p<0.05$) or similarities ($p\geq0.05$) between average concentration values over the total duration of the 725 d study, under the influence of two factors: 1) waste introduction method, and 2) time of operation, at a confidence level of 95%.

Values were compared cumulatively between all ADs (MX, ST, NO TP, UD), ADs that were mixed (MX, NO TP, UD), ADs that contained urine (MX, ST, NO TP), and between individual AD pairs to determine their statistical 'pairing' (similarity). Results from two-way ANOVA are shown below in Table S1.

Table S1. Results of ANOVA testing for analyzed constituents of anaerobic digester supernatant

	**pH** *(n=3)*			**Electrical conductivity** *(n=3)*		
	**F**	**p**	**Paired?**	**F**	**p**	**Paired?**
All ADs	835	6 E-161		276	1 E-89	
Mixed ADs	988	6 E-116		396	1 E-73	
Urine ADs	454	6 E-82		1.19	0.31	•
MX, NO TP	11.7	9 E-4		2.40	0.12	•
MX, UD	1,070	3 E-61		392	5 E-38	
MX, ST	755	2 E-53		0.94	0.34	•
NO TP, ST	528	2 E-45		1.41	0.24	•
NO TP, UD	1,056	5 E-61		402	2 E-38	
ST, UD	551	2 E-46		223	3 E-28	
**Remarks:**	No statistical pairing. Closest pairing are mixed ADs with urine.			ADs with urine cumulatively and individually paired.		
	**Chemical oxygen demand** *(n=4)*			**Dissolved organic carbon** *(n=1)*		
	**F**	**p**	**Paired?**	**F**	**p**	**Paired?**
All ADs	51.3	4 E-23		31.3	2 E-9	
Mixed ADs	72.7	2 E-20		8.00	3 E-3	
Urine ADs	2.99	0.06	•	30.5	9 E-7	
MX, NO TP	2.80	0.10	•	1.29	0.28	•
MX, UD	120	4 E-15		12.4	6 E-3	
MX, ST	2.23	0.14	•	44.1	6 E-5	
NO TP, ST	3.35	0.07	•	64.0	1 E-5	
NO TP, UD	65.1	1 E-10		10.7	8 E-3	
ST, UD	80.9	4 E-12		62.2	1 E-5	
**Remarks:**	ADs with urine cumulatively and individually paired.			MX and NO TP paired (mixing with urine).		
	**Total ammoniacal nitrogen** *(n=4)*			**Total phosphorous** *(n=4)*		
	**F**	**p**	**Paired?**	**F**	**p**	**Paired?**
All ADs	259	4 E-66		249	2 E-65	
Mixed ADs	263	3 E-45		265	7 E-46	
Urine ADs	43.9	4 E-15		21.0	1 E-8	
MX, NO TP	0.21	0.65	•	27.1	2 E-6	
MX, UD	301	2 E-25		269	2 E-24	
MX, ST	67.2	2 E-11		29.3	1 E-6	
NO TP, ST	45.1	7 E-9		1.74	0.19	•
NO TP, UD	274	2 E-24		289	3 E-25	
ST, UD	380	4 E-28		278	8 E-25	

**Remarks:**	MX and NO TP paired (mixing with urine).			NO TP and ST paired (less toilet paper in supernatant).		
	**Total dissolved solids** *(n=1)*			**Total suspended solids** *(n=1)*		
	**F**	**p**	**Paired?**	**F**	**p**	**Paired?**
All ADs	57.0	8 E-16		10.9	1 E-5	
Mixed ADs	70.7	2 E-12		4.98	0.01	
Urine ADs	8.63	1 E-3		12.3	1 E-4	
MX, NO TP	23.2	2 E-4		1.41	0.25	•
MX, UD	70.8	3 E-7		7.13	0.02	
MX, ST	1.42	0.25	•	16.6	9 E-4	
NO TP, ST	6.03	0.03		29.1	6 E-5	
NO TP, UD	83.4	1 E-7		9.8	6 E-3	
ST, UD	58.7	1 E-6		14.7	1 E-4	
**Remarks:**	MX and ST paired.			MX and NO TP paired (mixing with urine).		
	**% Volatile solids/total solids** *(n=1)*			***E. coli*** *(n=1)*		
	**F**	**p**	**Paired?**	**F**	**p**	**Paired?**
All ADs	162	4 E-25		29.6	5 E-5	
Mixed ADs	213	3 E-19		28.1	9 E-4	
Urine ADs	3.08	0.06	•	1.60	0.28	•
MX, NO TP	0.03	0.87	•	1.37	0.33	•
MX, UD	198	2 E-10		31.2	0.01	
MX, ST	3.39	0.08	•	1.92	0.26	•
NO TP, ST	5.08	0.04		1.61	0.29	•
NO TP, UD	345	3 E-12		26.0	0.01	
ST, UD	196	2 E-10		35.4	0.01	
**Remarks:**	ADs with urine cumulatively paired, MX individually paired with other urine-containing ADs. NO TP and ST are not paired, but close.			ADs with urine cumulatively and individually paired.		

2.2. Spearman's correlation

A Spearman's correlation was used to determine relationships between the trends observed in concentration data over the 725 d study. Concentration values were ranked according to their relative position within the overall data set, and correlation coefficients were obtained and characterized as: no or very low (VL, $\rho \geq 0$-0.3), low (L, $\rho \geq 0.3$-0.5), moderate (M, $\rho \geq 0.5$-0.7), high (H, $\rho \geq 0.7$-0.9), and very high (VH, $\rho \geq 0.9$), as shown in Table S2.

Table S2. Spearman's correlation for analyzed constituents of anaerobic digester supernatant

			**pH**			
**ADs**	**MX, NO TP**	**MX, ST**	**MX, UD**	**NO TP, ST**	**NO TP, UD**	**ST, UD**
**ρ**	0.911	0.923	0.443	0.784	0.318	0.404
**Correlation**	VH	VH	L	H	L	L
**Remarks:**	Trends in pH are strongly affected by the presence of urine.					
			**Electrical conductivity (EC)**			
**ρ**	0.997	0.913	0.268	0.910	0.275	-0.141
**Correlation**	VH	VH	VL	VH	VL	VL
**Remarks:**	Trends in EC are strongly affected by the presence of urine.					
			**Chemical oxygen demand (COD)**			
**ρ**	0.889	0.849	0.672	0.639	0.648	0.499
**Correlation**	H	H	M	M	M	L

**Remarks:**	Trends in COD are affected by the presence of urine, and less in absence of toilet paper and urine.					
**Dissolved organic carbon (DOC)**						
**ρ**	0.788	0.753	0.443	0.887	0.526	0.625
**Correlation**	H	H	L	H	M	M
**Remarks:**	Trends in DOC are affected by the presence of urine.					
**Total ammoniacal nitrogen (TAN)**						
**ρ**	0.922	0.882	0.528	0.838	0.460	0.615
**Correlation**	VH	H	M	H	L	M
**Remarks:**	Trends in TAN are affected by the presence of urine, and higher with mixing.					
**Total phosphorous (P_{tot})**						
**ρ**	0.957	0.939	0.337	0.948	0.464	0.294
**Correlation**	VH	VH	L	VH	L	VL
**Remarks:**	Trends in P_{tot} are strongly affected by the presence of urine.					
**Total dissolved solids (TDS)**						
**ρ**	0.966	0.932	0.851	0.907	0.888	0.808
**Correlation**	VH	VH	H	VH	H	H
**Remarks:**	Trends in TDS are strongly affected by the presence of urine, and similar without urine.					
**Total suspended solids (TSS)**						
**ρ**	0.442	0.448	0.345	0.475	0.596	0.821
**Correlation**	L	L	L	L	M	H
**Remarks:**	Trends in TSS are affected by the absence of mixing *and* the presence of toilet paper.					
**% Volatile solids/total solids (%VS/TS)**						
**ρ**	0.607	0.410	0.018	0.457	0.382	-0.244
**Correlation**	M	L	VL	L	L	VL
**Remarks:**	Trends in %VS/TS are slightly affected by the presence mixing *and* the presence of urine.					
E. coli						
**ρ**	0.998	0.417	-0.237	0.442	-0.249	0.606
**Correlation**	VH	L	VL	L	VL	M
**Remarks:**	Trends in *E. coli* are strongly affected by the presence of mixing *and* the presence of urine.					

3. Characteristics of anaerobic digestate

3.1. Digestate appearance

The visual appearance of digestate differed between the anaerobic digesters as a result of feeding and mixing regimes and changed over time with concentration of the digestate in response to repeat waste introduction (Fig. S4). NO TP and MX were the most similar in appearance and odor, which aligned with their statistically paired chemical characteristics. Settling in ST was apparent, and the rust-colored liquid digestate layer darkened in hue over the duration of the study, while the size of particles in the sludge layer decreased toward the end of the study.

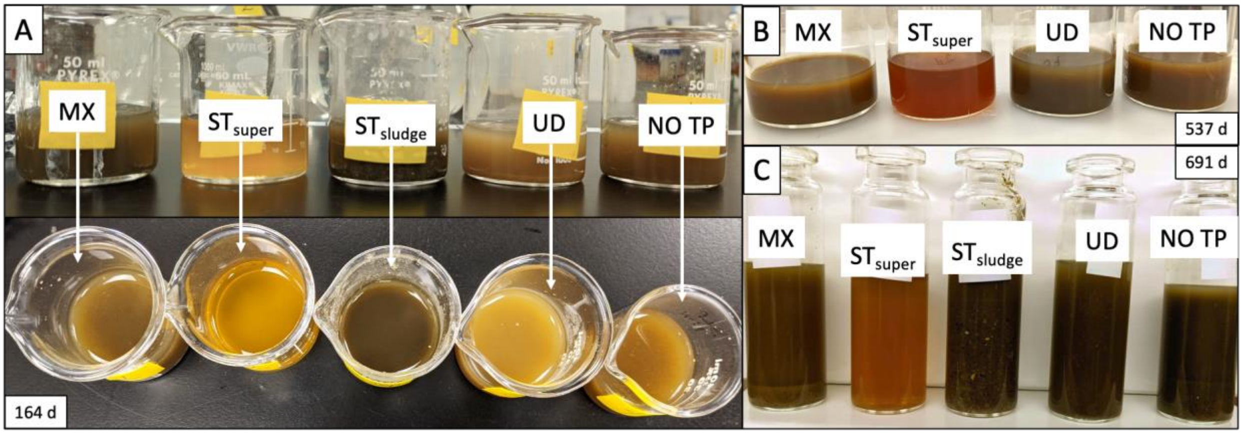

Figure S4. Visual appearance of digestate drawn from ADs (mixed = MX, unmixed/static = ST, urine diversion = UD, tissue exclusion = NO TP) on days A) 164, B) 537, and C) 691. A and C include samples of sludge from bottom layer of unmixed (ST) tank.

3.2. Fungal growth

Fungal overgrowth was first observed on the surface of the urine diversion tank (UD) after ~347 d of operation (Fig. S6), when a thick, rigid layer of white mold formed a barrier over the air-exposed supernatant surface. The growth was removed to eliminate impedance to the showering effect of the agitator pumps and reappeared at least three more times throughout the remaining duration of the study.

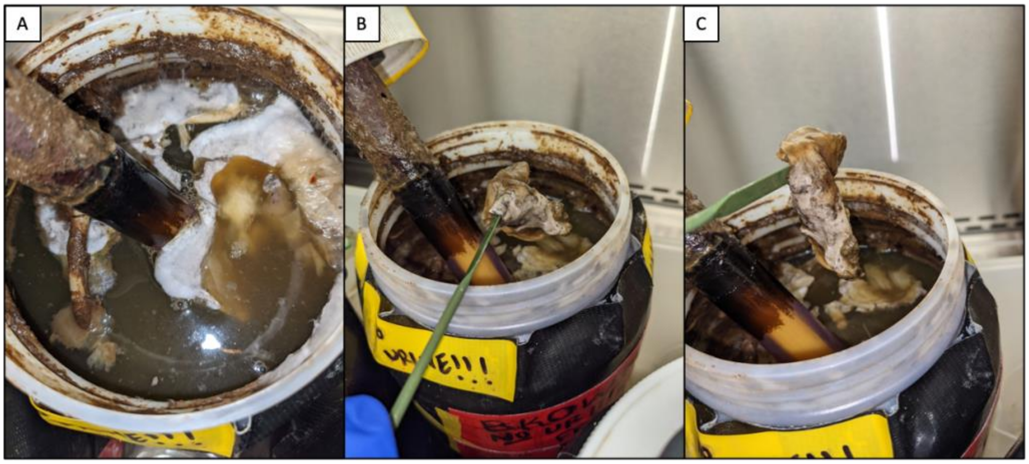

Figure S6. A) Fungal growth in urine-diverting anaerobic digester. Gas bubbles from anaerobic digestion are visible between broken segments of fungus. B-C) Thickness and rigidity of fungal formation upon removal from anaerobic digester.

3.3. Formation of ammonia odor

HACH Test-n-Tube (TNT) methods were used in this study to quantify total ammoniacal nitrogen (TAN) present in the AD digestate, a combination of ammonia (NH_3) and ammonium (NH_4^+). It is well known that NH_3 and NH_4^+ equilibrate in a mixture as a function of pH. Eqn. S1 was used to determine the theoretical fraction of NH_3 present in the digestate based on the pH throughout the duration of the study. The pH-dominated equilibrium of NH_3/NH_4^+ was determined according to:

$$K_a = \frac{[NH_3][H^+]}{[NH_4^+]} = 10^{-9.26} \rightarrow \quad NH_3 \text{ fraction} = \frac{1}{1+10^{(9.26-pH)}} \qquad \text{(Eqn. S1)}^{47}$$

The presence of ammonia odor was first detected at ~240 d of operation of ADs but may have been present prior to detection. This shift from sulfurous odor previously emitted from the ADs is attributed to the increasing fraction of NH_3, which is known to heighten the likelihood of ammonia volatilization[48]. Volatilization of NH_3 may also serve as an explanation for the losses in TAN (Fig. S5) that appeared for MX and NO TP, with the highest theoretical fraction of NH_3.

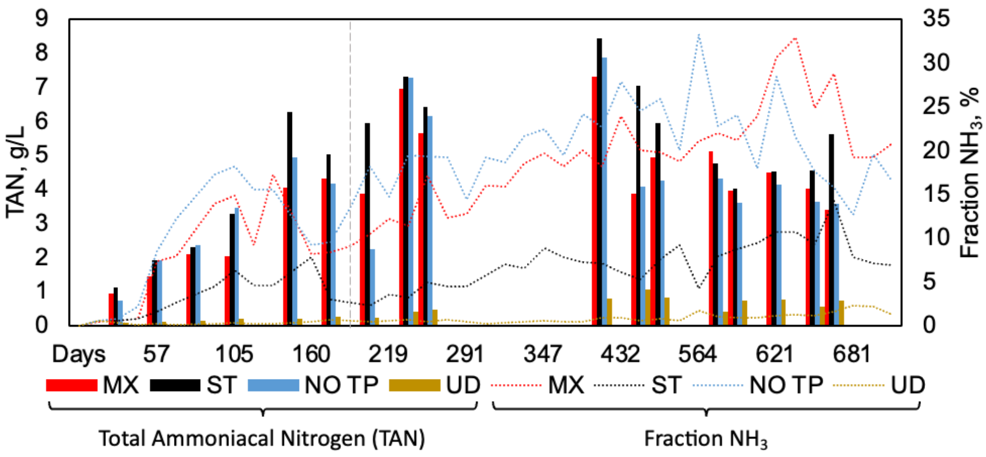

Figure S5. Measured total ammoniacal nitrogen (TAN, bars) vs theoretical fraction of NH_3 (lines) based on measured pH over 725 d study duration. Initial detection of ammonia odor at day 240 is denoted by gray dashed line.

4. Bacterial characteristics of digestate

4.1. Bacterial enumeration methods and digestate heterogeneity

Bacterial enumeration was performed at several stages of the study, which proved difficult to quantify during mid-late stages. Enumeration was performed at multiple dilutions for each sample in many instances, and, where readings were inconclusive, repeat samples of digester supernatant were taken on sequential dates (≤7 d apart) seeking better results. Prior to analysis for month 18, enumeration methods were changed from IDEXX Colilert-18 to Colisure to alleviate issues that may have been caused by high sample color. However, results continued to present a high margin of error. Figures S7 and S8 include all readings for total coliforms (TC) and *E. coli* respectively, and Figure S9 shows results from IDEXX heterotrophic plate counts (HPC).

We hypothesize that this issue was encountered due to the formation of heterogeneous flocs of bacteria and organic matter within the mixture, as increasing the number of sample location points within the supernatant and sludge, along with thorough mixing of the samples and subsequent dilutions, did not yield improved results. The *E. coli* values reported in the main text were based on the following: 1) one valid result at lower dilution; 2) average of two dilutions with valid results when the lower dilution was significantly close to the maximum; 3) average of two dilutions when both valid results were significantly close to the minimum. During month 23, where low dilution TC and *E. coli* trays were empty (<1) and higher dilution trays were full (>2,419.6) bacterial counts were not reported. In cases where *E. coli*>TC, results did not meet quality control standards, and were thus removed from the data set.

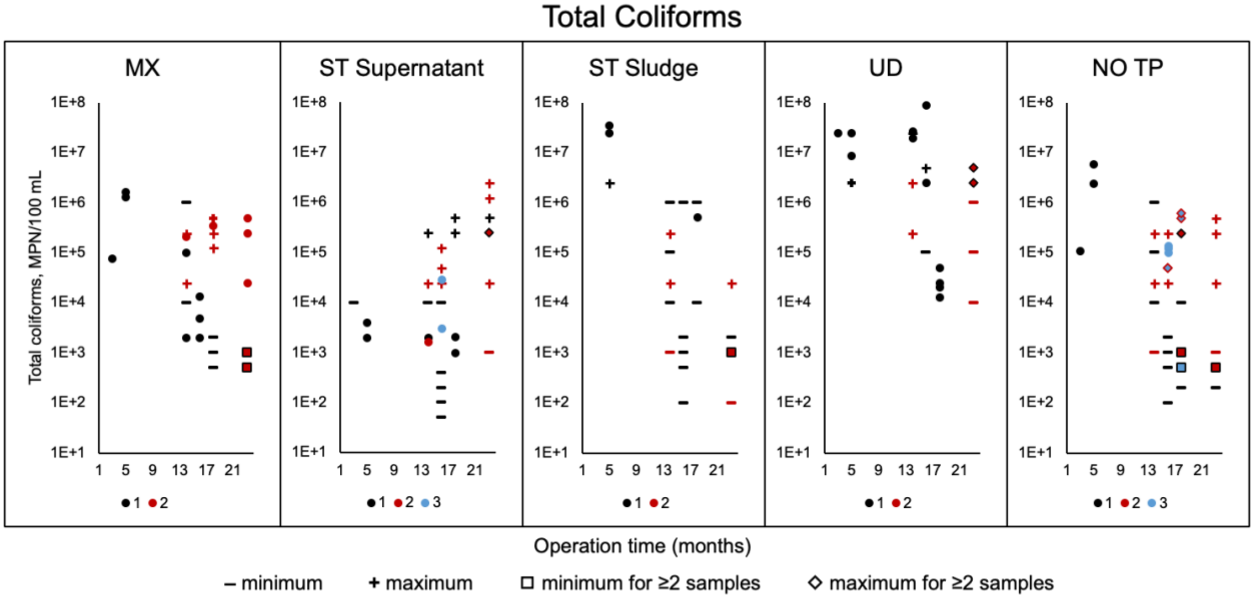

Figure S7. IDEXX Quanti-tray readings for total coliforms in anaerobic digesters under mixed (MX), unmixed (ST) supernatant and sludge, mixed toilet paper exclusion (NO TP) and mixed urine diversion (UD) waste introduction regimes. Repeat samples on sequential dates are shown as 1) black, 2) red, and 3) blue, where applicable. Minimum values (–) indicate that trays were empty, and results were <1 for their respective dilution. Maximum values (+) indicate that trays were full, and results were >2,419.6 for their respective dilution. Repeat dilutions on sequential dates that both resulted in a minimum or maximum are shown as ☐ and ◇, respectively.

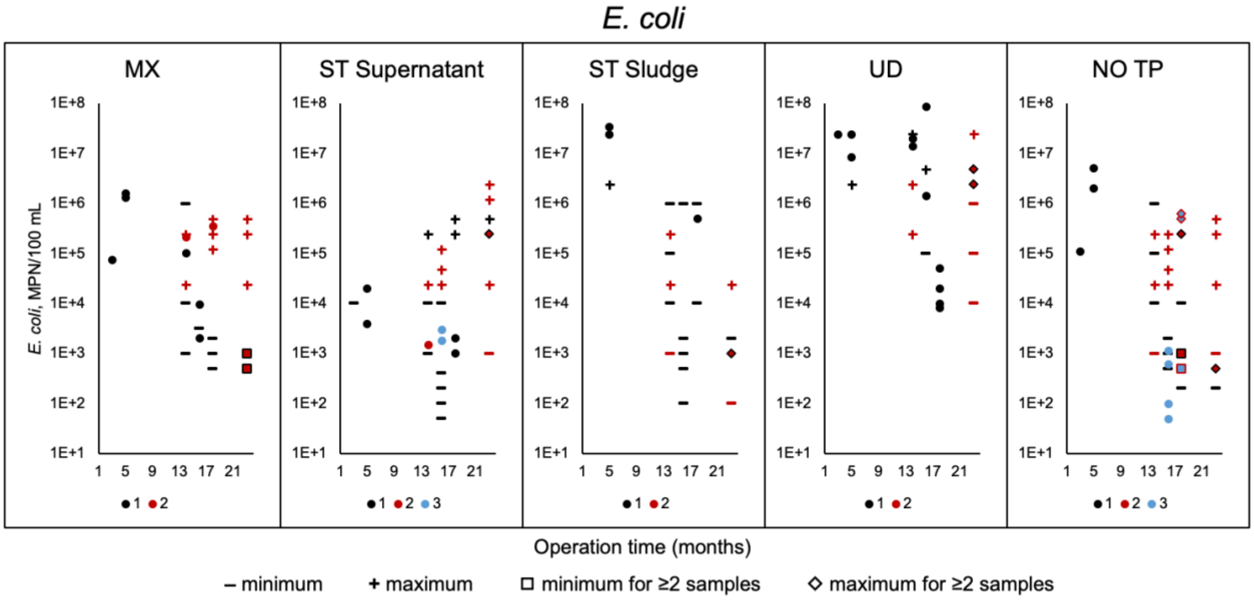

Figure S8. IDEXX Quanti-tray readings for *E. coli* in anaerobic digesters under mixed (MX), unmixed (ST) supernatant and sludge, mixed toilet paper exclusion (NO TP) and mixed urine diversion (UD) waste introduction regimes. Repeat samples on sequential dates are shown as 1) black, 2) red, and 3) blue, where applicable. Minimum values (–) indicate that trays were empty, and results were <1 for their respective dilution. Maximum values (+) indicate that trays were full, and results were >2,419.6 for their respective dilution. Repeat dilutions on sequential dates that both resulted in a minimum or maximum are shown as □ and ◇, respectively.

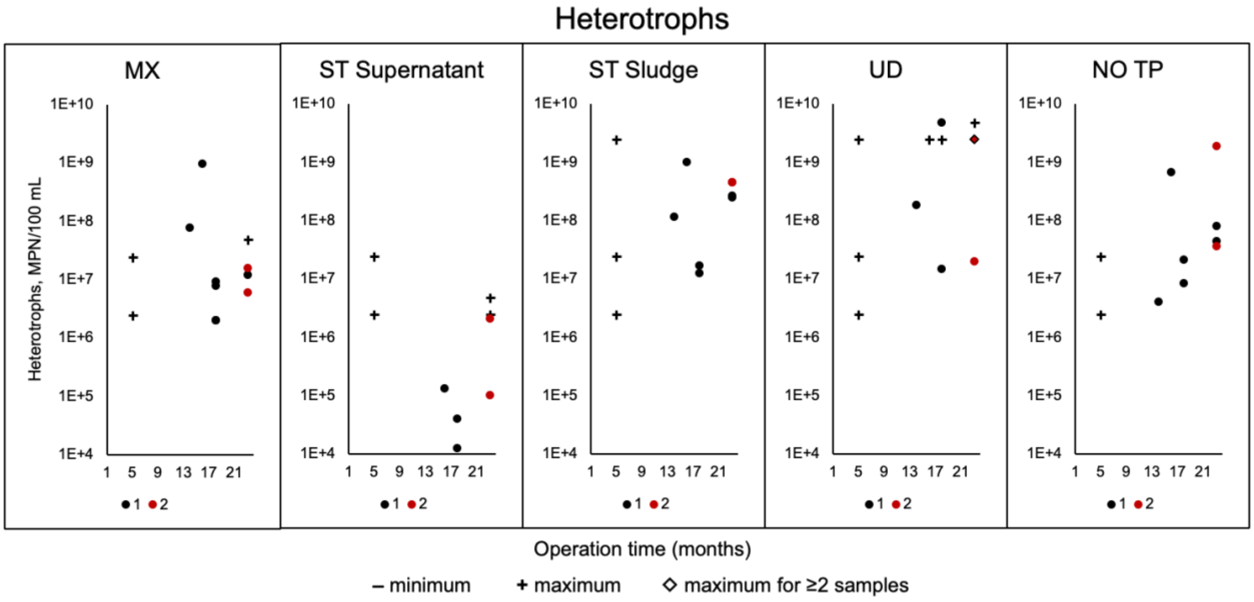

Figure S9. IDEXX Quanti-tray readings for heterotrophic plate counts (HPC) in anaerobic digesters under mixed (MX), unmixed (ST) supernatant and sludge, mixed toilet paper exclusion (NO TP) and mixed urine diversion (UD) waste introduction regimes. Repeat samples on sequential dates are shown as 1) black and 2) red. Minimum values (–) indicate that trays were empty, and results were <1 for their respective dilution. Maximum values (+) indicate that trays were full, and results were >2,419.6 for their respective dilution. Repeat dilutions on sequential dates that both resulted in a maximum are shown as ◇. Dilutions for each sample are not displayed on charts for brevity.

References

1. World Health Organization (WHO) & the United Nations Children's Fund (UNICEF). Progress on household drinking water, sanitation and hygiene 2000-2020: Five years into the SDGs. (2021).
2. Trimmer, J. T. et al. (2020). Navigating multidimensional social-ecological system trade-offs across sanitation alternatives in an urban informal settlement. *Environ Sci. Technol.*, **54**, 12641-12653. (2020).
3. Graham, J. P. & Polizzotto, M. L. Pit Latrines and their impacts on groundwater quality: a systematic review. *Environ. Health. Perspec.*, **121(5)**, pp. 521-530. (2013).
4. Nakagiri, A., et al. Are pit latrines in urban areas of Sub-Saharan Africa performing? A review of usage, filling, insects and odour nuisances. *BMC Public Health*, **16(120)**. (2016).
5. Colón, J., Forbis-Stokes, A. & Deshusses, M. Anaerobic digestion of undiluted simulant human excreta for sanitation and energy recovery in less-developed countries. *Energy Sustain. Dev.*, **29**, pp. 57-64. (2015).
6. Kumwenda, S., Msefula, C., Ngwira, B., Morse, T. & Ensink, J. H. J. Knowledge, attitudes and practices on use of fossa alternas and double vault urine diverting dry (DVUDD) latrines in Malawi. *J. Water Sanit. Hyg. Dev.*, **6(4)**, pp. 555-568. (2016).
7. Temesgen, A., Adane, M. M., Birara, A. & Shibabaw, T. Having a latrine facility is not a guarantee for eliminating open defecation owing to socio-demographic and environmental factors: The case of Machakel district in Ethiopia. *PLoS One*, **16(9)**, 0257813. (2021).
8. Paterson, C., Mara, D. & Curtis, T. Pro-poor sanitation technologies. *Geoforum*, **38(5)**, pp. 901-907. (2007).
9. Rajagopal, R., Massé, D. I. & Singh, G. A critical review on inhibition of anaerobic digestion process by excess ammonia. *Bioresour. Technol.*, **143**, pp. 632-641. (2013).
10. Poirer, S., Quéméner, E. D., Madigou, C., Bouchez, T. & Chapleur, O. Anaerobic digestion of biowaste under extreme ammonia concentration: Identification of key microbial phylotypes. *Bioresour. Technol.*, **207**, pp. 92-101. (2016).
11. Bakare, B.F., Foxon, K. M., Brouckaert, C. J. & Buckley, C. A. Variation in VIP latrine sludge contents. *Water SA*, **38(4)**, pp. 479-486. (2012).

12. Buckley, C. A. et al. Scientific support for the design and operation of ventilated improved pit latrines. WRC Report No. **TT 357/08**. Water Research Commission, Pretoria.
13. Changara, M. C., Sanyika, W. T., Bangira, C. & Misi, S. Physico-chemical properties and bacterial community structure dynamics during the mesophilic anaerobic digestion of pit latrine faecal sludge. *Water SA*, **45(3)**, pp. 338-348. (2019).
14. Carroll, Z. S. & Long, S. C. Bench-scale analysis of surrogates for anaerobic digestion processes. *Water Environ. Res.*, **88(5)**, pp. 458-467. (2016).
15. Manser, N. D., Mihelcic, J. R. & Ergas, S. J. Semi-continuous mesophilic anaerobic digester performance under variations in solids retention time and feeding frequency. *Bioresour. Technol.*, **190**, pp. 359-366. (2015).
16. Zuma, L., Velkushanova, K. & Buckley, C. Chemical and thermal properties of VIP latrine sludge. *Water SA*, **41(4)**, pp. 534-540. (2015).
17. Jegede, A. O., Zeeman, G. & Bruning, H. Effect of mixing regimes on cow manure digestion in impeller mixed, unmixed, and Chinese dome digesters. *MDPI Energies*, **12(2450)**, pp. 1-14. (2019).
18. Almomani, F. Field study comparing the effect of hydraulic mixing on septic tank performance and sludge accumulation. *Environ. Tech.*, **37(5)**, pp. 521-534. (2014).
19. Larsen, T. A., Gruendl, H. & Binz, C. The potential contribution of urine source separation to the SDG agenda – a review of the progress so far and future development options. *Water Res. Technol.*, **7**, pp. 1161-1176. (2021).
20. Malila, R., Lehtoranta, S. & Viskari, E.-L. The role of source separation in nutrient recovery – Comparison of alternative wastewater treatment systems. *J. Cleaner Prod.*, **219**, pp. 350-358. (2019).
21. Boyer, T. H. & Saetta, D. Opportunities for building-scale urine diversion and challenges for implementation. *Acc. Chem. Res.*, **52**, pp. 886-895. (2019).
22. Chaggu, E. J., Sanders, W. & Lettinga, G. Demonstration of anaerobic stabilization of black water in accumulation systems under tropical conditions. *Bioresour. Technol.*, **98**, pp. 3090-3097. (2007).
23. Coelho, L. P. et al. Similarity of the dog and human gut microbiomes in gene content and response to diet. *Microbiome*, **6(72)**, (2018).

24. Penn, R., Ward, B. J., Strande, L. & Maurer, M. Review of synthetic human faeces and faecal sludge for sanitation and wastewater research. *Water Res.*, **132**, pp. 222-240. (2018).

25. Pronk, W., Palmquist, H., Biebow, M. & Boller, M. Nanofiltration for the separation of pharmaceuticals from nutrients in source-separated urine. *Water Res.*, **40**, pp. 1405-1412. (2006).

26. World Health Organization (WHO). A guide to the development of on-site sanitation. (1992).

27. Rose, C., Parker, A., Jefferson, B. & Cartmell, E. The characterization of feces and urine: A review to inform advanced treatment technology. *Crit. Rev. Env. Sci. Technol.*, **45(17)**, pp. 1827-1879. (2015).

28. Toilet Paper History. Toilet paper fun facts. http://www.toiletpaperhistory.net/toilet-paper-facts/toilet-paper-fun-facts/. Accessed 2020.

29. American Public Health Association (APHA). Standard methods for the examination of water and wastewater. **23rd ed.** (2017).

30. Vesvikar, M., & Al-Dahhan, M. Effects of mixing and scale on the performance and hydrodynamics of anaerobic digesters. *ECI Symposium Series*, Bioenergy – I: From concept to commercial processes. (2006).

31. Yenigün, O. & Demirel, B. Ammonia inhibition in anaerobic digestion: a review. *Process Biochem.*, **48**, pp. 901-911. (2013).

32. Sievers, D. M. & Brune, D.E., Carbon/Nitrogen ratio and anaerobic digestion of swine waste. *Trans. ASAE*, **3**, pp. 537-541. (1978).

33. Ellouze, M., Aloui, F. & Sayadi, S. Effect of high ammonia concentrations on fungal treatment of Tunisian landfill leachate. *Desalination*, **248**, pp. 147-156. (2008).

34. Lindmark, J., Thorin, E., Fdhila, R. B. & Dahlquist, E. Effects of mixing on the result of anaerobic digestion: Review. *Renewable Sustainable Energy Rev.*, **40**, pp. 1030-1047. (2014).

35. Jiang, Y. et al. Ammonia inhibition and toxicity in anaerobic digestion: a critical review. *J. Water Process Eng.*, **32(100899)**, pp. 1-34. (2019).

36. Bonmatí, A., Flotats, X., Mateu, L. & Campos, E. Study of thermal hydrolysis as a pretreatment to mesophilic anaerobic digestion of pig slurry. *Water Sci. Technol.*, **44(4)**, pp. 109-116. (2001).
37. Ribeiro, T. et al. Underestimation of dry matter of anaerobic media with high bicarbonate concentration. *Appl. Sci.*, **12(3)**, 1105. (2022).
38. Carrère, H., Sialve, B. & Bernet, N. Improving pig manure conversion into biogas by thermal and thermo-chemical pretreatments. *Bioresource Technology*, **100**, pp. 3690-3694. (2009).
39. Yu, T. et al. Effect of alkaline microwaving pretreatment on anaerobic digestion and biogas production of swine manure. *Sci. Rep.*, **7(1688)**, pp. 1-8. (2017).
40. Mao, C., Feng, Y., Wang, X. & Ren, G. Review on research achievements of biogas from anaerobic digestion. *Renewable Sustainable Energy Rev.*, **45**, pp. 540-555. (2015).
41. Cerón-Vivas, A., Cáceres, K. T., Rincón, A. & Cajigas, Á. A. Influence of pH and C/N ratio on the biogas production of wastewater. *Revista facultad de ingeniería*, **92**, pp. 70-79. (2019).
42. Mamais, D., Pitt, P. A., Cheng, Y. W., Loiacono, J., & Jenkins, D. Determination of ferric chloride dose to control struvite precipitation in anaerobic sludge digestion. *Water Environ. Res.*, **66(7)**, pp. 912-918. (1994).
43. Yu, C., Yin, W., Yu, Z., Chen, J., Huang, R. & Zhou, X. Membrane technologies in toilet urine treatment for toilet urine resource utilization: a review. *RSC Adv.*, **11(56)**, pp. 35525-35535. (2021).
44. Khan, M. N., Lacroix, M. & Wessels, C. Converting wastewater cellulose to valuable products: A techno-economic assessment. *J. Clean. Prod.*, **365**, 132812. (2022).
45. Chen, R., Nie, Y., Kato, H., Wu, J., Utashiro, T., Lu, J., Yue, S., Jiang, H., Zhang, L. & Li, Y.-Y. Methanogenic degradation of toilet-paper cellulose upon sewage treatment in an anaerobic membrane bioreactor at room temperature. *Bioresour. Technol.*, **228**, pp. 69-76. (2016).
46. Du, H. & Li, F. Characteristics of dissolved organic matter formed in aerobic and anaerobic digestion of excess activated sludge. *Chemosphere*, **168**, pp. 1022-1031. (2017).

47. Freguia, S., Sharma, K., Benichou, O., Mulliss, M. & Shon, H. K. Sustainable engineering of sewers and sewage treatment plants for scenarios with urine diversion. *J. Hazard. Mater.*, **415**, 125609. (2021).

48. Masters, G. M. & Ela, W. P. Introduction to Environmental Engineering and Science, 3rd ed. Prentice Hall. (2007).

49. Koirala, K. et al. Impact of anaerobic digestion of liquid dairy manure on ammonia volatilization process. *Transactions of the ASABE*, **56(5)**, pp. 1959-1966. (2013).

50. DeVilbiss, S. E., Steele, M. K., Krometis, L. H. & Badgley, B. D. Freshwater salinization increases survival of *Escherichia coli* and risk of bacterial impairment. *Water Res.*, **191**, 116812. (2021).

51. Samer, M. Biological treatment processes. In *Wastewater Treatment Engineering*; Intech: London, UK, (2015).

52. Republic of South Africa Department of Water Affairs and Forestry (DWAF). Options for dealing with fill pits – Draft guideline – emptying of pit latrines. *Ministry of Water Affairs (DWAF).* (2005).
